# The quality of life of the patients with diabetes type 2 using EQ-5D-5 L in Birjand

**DOI:** 10.1186/s12955-020-1277-8

**Published:** 2020-01-30

**Authors:** Mohammad Reza Abedini, Bita Bijari, Zahra Miri, Fatemeh Shakhs Emampour, Ali Abbasi

**Affiliations:** 1 Cellular and Molecular Research Center, Birjand University of Medical Sciences (BUMS), Birjand, Iran; 2 Cardiovascular Diseases Research Center, Community Medicine, Birjand University of Medical Sciences, Birjand, Iran; 3 Birjand University of Medical Sciences, Birjand, Iran; 4 Anesthesiology, Department of Anesthesia, Birjand University of Medical Sciences, Birjand, Iran; 5 Medical education, Birjand University of Medical Sciences, Birjand, Iran

**Keywords:** Quality of life, Diabetes type 2, EQ-5D-5 L

## Abstract

**Background:**

Due to high prevalence of diabetes and its complications, evaluating of the patients’ quality of life is critical. EQ-5D-5 L is a valid tool for assessing the quality of life in chronic diseases including diabetes. The present study conducted to illustrate the quality of life for the patients who referred to the Diabetes clinic and determine its relationship with their demographic and clinical characteristics in Birjand in 2017.

**Methods:**

In this cross-sectional study, 300 patients with type 2 diabetes were selected through a systematic sampling in 2017. Data were collected using a checklist including patients’ demographic, clinical and laboratory information and the EQ-5D-5 L. Data were entered the SPSS (22) software, analyzed by independent sample T-test, ANOVA, Chi-Square and logistic regression tests. Statistical significance was inferred at α = 0.05.

**Results:**

Mean age for the participants was 58.1 ± 9.6 years. The mean score for the quality of life based on the EQ-5D-5 L and VAS scale were 0.89 ± 0.13 and 65.22 ± 9.32, respectively. Moderate and severe problems were found in the anxiety/depression dimensions in 12% of the patients, while these numbers for the presence of pain/discomfort and mobility were slightly higher (13.7 and 13.6%, respectively). The mean scores for quality of life and VAS were significantly higher in the men, employed and patients < 50 years age.

**Conclusion:**

The quality of life for the type 2 diabetes patients is affected by numerous factors including sex, occupation, duration of the disease and the presence of complications such as neuropathy and nephropathy.

## Background

In parallel with economy development, life standards improvement, lifestyle/diet changes and urbanization, non-communicable diseases like diabetes mellitus (DM) are the most important public health problems worldwide [1].

The prevalence of DM is increasing in the developed and developing countries. WHO reported that the number of diabetic patients in the world has increased from 110 million in 1994 to 240 million in 2010 and it is estimated to raise at 300 million in 2025 [2].

In Iran, the prevalence of DM is relatively high and has been estimated by various studies 12.4% in individuals aged 15–75 [2], 12.6% in aged group 40–64 [3], and 24.5% in the people aged 40–80 years old [4].

As with any other chronic disease, DM is associated with many personal, familial, social and financial issues and even higher mortality rate. Problems such as increased blood glucose, dietary and exercise limitation repeatedly demand for insulin injection, musculoskeletal complications, physical disabilities, sexual dysfunction and vascular disorders are some examples which negatively affect the lives of patients with DM [5].

Moreover, job loss, frequent hospitalization, higher demand for medical and patient care, indirect costs related to early death, reduced social and familial interactions, and worsening in lifestyle are some of the major problems which affect the familial, social and economic status of these patients [6].

In Iran, National Program for Prevention and Control of Diabetes has been introduced in the health system in 2004. Several levels of health care have been designed including the primary level in which health workers (the behvarz) in the health house and health technician in the urban health post perform the population evaluation and screening for DM. At the secondary level such as rural and urban health centers, general practitioners and laboratory technicians serve as the diabetes team members in this regard [7].

In the Diabetes clinic as a secondary level, several services are provided as follow: diagnosis, treatment and patients’ care, patients’ referral to the diabetes center, follow up feedback and appropriate action, assessing for complications according to clinical guidelines, and collecting as well as recording the patient information in the medical records [7].

Health-related quality of life (HRQoL) is one of the most widely measured treatment outcomes to self-assess the effects of the management of chronic disease on health, and monitors the physical, psychological and social aspects of personal health. It is influenced by individual expectations, beliefs, perceptions and experiences [8].

Numerous studies indicated that QoL for patients with DM is lower than that of the healthy individuals, and the factors involved in this regard are not precisely determined. It is noteworthy that some variables such as the type of DM, use of insulin, age, DM related complications, social status, psychological factors, ethnicity, educational level, knowledge about the disease, type of assistance which they received from others may interfere in the QoL for these patients [9].

So far several tools have been devised to assess the QoL including SF-36 tool [10] and EQ-5D created by Brook in 1991 [11]. The EQ-5D is one of the most feasible tools to assess individuals’ QoL, and evaluates their physical, mental and social performance [12]. It has been validated and used in many studies to determine QoL in chronic diseases such as diabetes, chronic lung diseases, stroke and chronic mental illnesses [13–17]. Currently three versions of EQ-5D are available including EQ-5D-3 L, EQ-5D-5 L and EQ-5D-y. The 5-level EQ-5D version (EQ-5D-5 L) was introduced by the EuroQol Group in 2009 to improve its sensitivity and reduce ceiling effects in comparison to EQ-5D-3 L [18].

EQ-5D-5 L is a short and clear questionnaire which could be easily completed in a short period of time by the patients, thereby substituted with the general quality of life questionnaire in epidemiological studies and clinical evaluation for diabetic patients.

The DM complications can be responsible for the most of morbidity and mortality associated with the disease. Therefore, assessing the patients’ QoL at regular intervals is a necessity for DM as a chronic disease. This evaluation, as a powerful tool, is critical in predicting patients’ status for disease management and long-term health care. Regular evaluation for QoL as a routine clinical practice could potentially improve necessary communication among the health care providers and their patients, thereby identify the complications and help them for long care resulting in improving their health status [19].

Evaluating the quality of life and its related factors can be helpful to improve the diabetic patients QoL. Due to the specific geographical and cultural characteristics of this region, QoL of the patients in this particular area and the factors affecting it may vary with other patients. Thereby using a short, brief and valid questionnaire which can be completed in a short time is beneficial for assessing patients’ QoL.

Therefore, the present study conducted to assess the QoL for patients with diabetes type 2 and its relationship with the demographic and clinical characteristics of these patients who referred to the Diabetes clinic in Birjand in 2017.

## Methods

### Study population

In this cross-sectional (descriptive analytical) study, 300 patients with type 2 diabetes who had a medical record in the Diabetes clinic in Birjand from September to December 2017 were selected in a systematic sampling. There were about 2000 medical records of diabetic patients in the Diabetes clinic in Birjand. The sample size was calculated based on the percentage of any reported problems in EQ-5D dimensions including mobility (MO), self-care (SC), usual activities (UA), pain/discomfort (P/D) and anxiety/depression (A/D). According to Javanbakht study [20], the patients reported “moderate or extreme problems” in different dimensions of the EQ-5D as follow: MO (30%), SC (24.6%), UA (32.9%), P/D (69.3%) and A/D (56.6%). The maximum sample size was calculated 295 patients considering the lowest percentages of reported problems. (Considering *p* = 24.6, 95% confidence level; and d = 0.05) using N = [z(1-α/2)^2^p(1-p)]/d^2^ formula. Then, we contacted them and provided a description of the study objective. Those patients with DM, who were interested to participate, were included in the study. Inclusion criteria were definitive diagnosis of DM and patient satisfaction for participation in the study.

### Measures

To collect the patients’ information, two tools have been used including a checklist containing demographic and clinical characteristics as well as their laboratory values. The second tool was EQ-5D-5 L which consisted of two-parts, the EQ-5D descriptive system and the EQ visual analogue scale (VAS) [18].

#### Scoring the EQ-5D-5 L descriptive system

The descriptive system comprises five dimensions such as mobility, self-care, usual activities, pain/discomfort and anxiety/depression. Each dimension ranked at 5 levels as follow: no problems (1), slight problems (2), moderate problems (3), severe problems (4), and extreme problems (5). The patient was asked to indicate his/her health status by selecting the most appropriate statement in each of the five dimensions. Each state is referred to a 5-digit code.

The digits for the five dimensions can be combined into a 5-digit number that describes the patient’s health state. For example, state 11,111 indicates no problems on any of the 5 dimensions, while state 12,345 indicates no problems with mobility, slight problems with washing or dressing, moderate problems with doing usual activities, severe pain or discomfort and extreme anxiety or depression. To convert an individual EQ-5D health state to a single summary index a value set is required. In the present study due to the absence of a locally appropriate set of values, as suggested by EuroQol Group, the EQ-5D score was calculated using standard value sets produced by the EuroQol Groups’ standardized valuation technology (EQ-VT) which considered 1 = highest QoL, and 0 = the least QoL [18]. According to EQ-5D-5 L user guide (18), one way for data presentation as a health profile is via a table including the frequency of reported problems for various levels of each dimension. It is Sometimes more convenient to dichotomies the EQ-5D-5 L levels into ‘no problems’ (i.e. level 1) and ‘problems’ (i.e. levels 2 to 5), therefore changing the profile into frequencies of reported problems (18). In this context, we have also changed the profile into frequencies for reported data.

#### Scoring the EQ-5D-5 L VAS

The EQ VAS records the patients’ self-rated health on a vertical visual analogue scale which was scored from zero to 100, where the endpoints are labeled as ‘The best health you can imagine’ and ‘The worst health you can imagine’. Indeed, visual scale of the VAS 100 means the best health status and 0 means the worst health status you can imagine [18].

#### Psychometric properties

Before gathering the patients’ information, the questionnaire was translated to Persian by a native Iranian health professional translator who was fluent in both English and Persian languages. Subsequently, the questionnaire was back translated to English. Then, two versions of the questionnaire compared by the investigators, so any possible variations between them were discussed and corrected accordingly.

Finally, the Persian version of the questionnaire was tested on few patients and results showed that all the patients easily understood the items.

Factor analysis was undertaken on the five dimensions of the EQ-5D-5 L. The analysis produced a single component which accounted for 60.99% of the variance indicative that the dimensions can be added together to create a single index score. Internal consistency was also assessed using the coefficient Cronbach’s α which was calculated 0.83 for this questionnaire. To test the construct validity of the EQ-5D-5 L, the SF-36 questionnaire was administered for the subjects, Pearson’s correlation coefficient was used to assess this type of validity and demonstrated that all the correlations were significant at the level of 0.01. The most powerful correlation was between pain/discomfort (P/D) of EQ-5D-5 L and pain of SF-36 (0.47).

### Data collection

The demographic checklist and EQ-5D-5 L questionnaire were handed into the patients and collected after completion in the clinic between 8 am and 12 pm. The detailed data for DM history, laboratory values, presence and type of complications were obtained from the medical records. The information from the illiterate patients was collected by the researcher after reading the questions for them. Diagnosis of diabetic complications including nephropathy and neuropathy as well as retinopathy was performed and recorded in the patients’ profile by an Internal Medicine specialist and an ophthalmologist, respectively.

### Statistical analysis

Data were entered into SPSS (22) software. After determining the normal distribution, data were analyzed with independent sample T-test, ANOVA, Chi-Square and logistic regression tests. Statistical significance was inferred at α = 0.05.

In logistic regression model, the dependent variable (QoL) in each domain from EQ-5D-5 L were dichotomized into ‘no problems’ (= level1) and ‘problems’ (= levels 2–5). After entering independent variables into regression model including age, gender, education level, occupation, duration of diabetes, HbA1c values, prescribed treatment, the presence of Nephropathy, Retinopathy, and Neuropathy, the history for diabetes related hospitalization and the history of ischemic heart disease (IHD), to summarize the data only those variables were reported which shown a significant relationship with a domain from EQ-5D-5 L.

## Results

### Population

A total of 300 diabetic patients with a mean age of 58.1 ± 9.6 participated in this study (ranged from 32 to 93 years old). As shown in Table 1, the majority were women 178 (59.3%), married 299 (99.6%), housewife 145 (48%), urban residents 279 (93%) and from the age group of 60 and older 125 (41.6%).

**Table 1:** Distribution of the patients’ demographic and clinical characteristics

characteristics		N(%)
sex	female	178 (59.3)
	male	122 (40.7)
Age group	≤50y	56 (18.7)
	51-60y	119 (39.7)
	> 60	125 (41.6)
marriage	single	1 (0.3)
	married	299 (99.7)
Job	Employed	77 (25.7)
	housewife	145 (48.3)
	others	78 (26)
Education	illiterate	58 (19.3)
	undergraduate	153 (51)
	postgraduate	89 (29.7)
Residency	Urban	279 (93)
	rural	21 (7)
treatment	Oral drug	228 (76)
	insulin	72 (24)
HbA1C	< 7	57 (19)
	≥7	243 (81)
duration	<10y	189 (63)
	≥10y	11 (37)
Neuropathy	yes	32 (10.7)
	no	268 (89.3)
Retinopathy	yes	83 (27.7)
	no	217 (72.3)
Nephropathy	yes	56 (18.7)
	no	244 (81.3)
IHD	yes	70 (23.3)
	no	300 (76.7)
Diabetes related hospitalization	yes	140 (46.7)
	no	160 (53.3)

### EQ-5D-5 L results

The mean score for the quality of life based on the EQ-5D-5 L questionnaire was 0.89 ± 0.13 (CI:0.87–0.90) and the mean score of VAS scale was 65.22 ± 9.32 (CI:64.16–66.23).

**Fig. 1:**
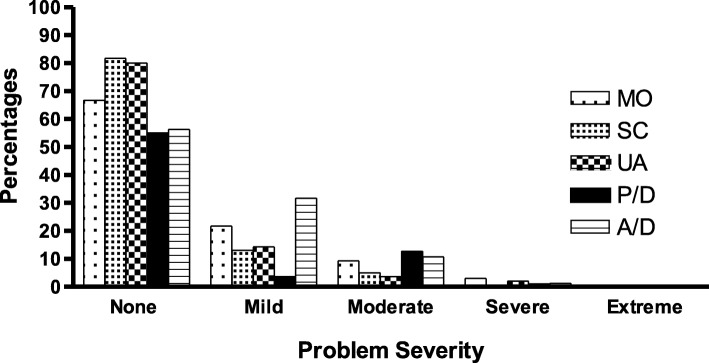
The problem severity (percentage of each level of problems) in 5 dimensions reported by the patients

Most of the patients did not report any problem or declared mild problems in various dimensions from the EQ-5D-5 L questionnaire. Higher percentages of patients indicated that they did not have any problem in different dimensions like mobility (65.7%), self-care (81.7%), usual daily activities (80%), the pain/discomfort (55%) and the anxiety/depression dimension (56.3%). However, moderate and severe problems were reported in some dimensions such as anxiety/depression (12%), pain/discomfort (13.7%) and mobility (13.6%). In Fig. 1, percentages of each level of problems are shown in 5 dimensions.

### Factors associated with QoL

Mean scores for the quality of life in men (0.92 ± 0.12) was significantly higher (*p* = 0.004) than that in women (0.86 ± 0.13). These scores for VAS scale were 68.85 ± 8.20 and 62.73 ± 9.25, respectively (*p* = 0.008).

The mean score for the QoL and the VAS scale were compared in Table 2 using patients’ demographic and clinical characteristics versus sex.

**Table 2:** Comparison the mean score for QoL and the VAS scale according to the patients’ demographic characteristics by sex

characteristics		ED-Q5			VAS		
		male	female	total	male	female	total
		Mean ± SD	Mean ± SD	Mean ± SD	Mean ± SD	Mean ± SD	Mean ± SD
Age group	≤50y	0.98 ± 0.03	0.91 ± 0.09	0. 93 ± 0.08	74.11 ± 6.11	66.41 ± 9.71	68.75 ± 9.54
	51-60y	0.95 ± 0.08	0.83 ± 0.15	0.87 ± 0.14	70.71 ± 9.72	61.18 ± 8.91	64.50 ± 10.24
	> 60	0.89 ± 0.14	0.86 ± 0.12	0.88 ± 0.13	66.19 ± 6.45	62.33 ± 8.76	64.28 ± 7.89
F		4.43	4.49	4.00	8.94	4.38	5.09
*P* value		0.01	0.01	0.01	< 0.001	0.01	0.007
Job	Employed	0.94 ± 0.11	0.93 ± 0.07	0.94 ± 0.10	70.93 ± 8.22	67.77 ± 11.27	70.19 ± 9.04
	housewife	–	0.87 ± 0.12	0.87 ± 0.12	–	62.38 ± 8.62	62.38 ± 8.62
	others	0.90 ± 0.13	0.72 ± 0.21	0.87 ± 0.16	63.90 ± 7.74	60.00 ± 11.01	65.57 ± 8.82
F or T		1.89	11.38	8.76	3.57	3.53	19.95
P value		0.06	< 0.001	< 0.001	0.002	0.03	< 0.001
Education Level	Illiterate	0.88 ± 0.13	0.87 ± 0.13	0.87 ± 0.13	66.87 ± 11.31	60.90 ± 8.37	61.72 ± 8.96
	undergraduate	0.91 ± 0.14	0.88 ± 0.10	0.89 ± 0.12	67.17 ± 7.16	63.63 ± 8.56	65.07 ± 8.18
	postgraduate	0.94 ± 0.08	0.80 ± 0.18	0.88 ± 0.15	71.15 ± 8.43	62.97 ± 11.63	65.75 ± 10.36
F		1.44	5.05	0.73	3.73	1.43	7.70
P value		0.24	0.007	0.47	0.02	0.24	0.001
Treatment type	Oral drug	0.93 ± 0.12	0.87 ± 0.13	0.89 ± 0.13	69.42 ± 8.46	63.12 ± 9.52	65.85 ± 9.46
	insulin	0.91 ± 0.11	0.83 ± 0.14	0.86 ± 0.13	66.85 ± 6.95	61.57 ± 8.37	63.55 ± 8.23
T		0.62	2.22	2.28	1.01	0.89	1.37
P value		0.53	0.02	0.02	0.30	0.36	0.16
HbA1C	< 7	0.96 ± 0.04	0.86 ± 0.16	0.89 ± 0.14	70.55 ± 7.25	63.84 ± 9.20	65.96 ± 9.13
	≥7	0.92 ± 0.13	0.86 ± 0.12	0.88 ± 0.13	68.55 ± 8.35	62.41 ± 9.27	65.06 ± 9.39
T		2.84	0.01	0.47	0.95	0.85	0.66
P value		0.006	0.99	0.63	0.30	0.39	0.50
DM history	<10y	0.95 ± 0.07	0.87 ± 0.12	0.90 ± 0.11	69.93 ± 7.78	63.91 ± 9.09	66.43 ± 9.42
	≥10y	0.88 ± 0.17	0.84 ± 0.14	0.85 ± 0.16	66.86 ± 6.63	60.80 ± 9.24	63.15 ± 8.81
T		2.4	1.61	2.83	2.00	2.20	2.98
P value		0.02	0.10	0.005	0.04	0.04	0.003

In terms of the age groups and irrespective of the sex, the mean scores for QoL and VAS were significantly higher for the patients younger than 50 years old compared to the other age groups (*p* < 0.05).

The QoL and VAS in the employed women and men were significantly higher than those for housewives, unemployed women and men (p < 0.05).

Mean scores of the QoL was not different in terms of the education level in all individuals (*p* = 0.47) and also in the men (*p* = 0.24); however, in the illiterate and the undergraduate women, it was significantly higher than postgraduate women (*p* = 0.007). While, VAS mean scores were significantly higher in postgraduate than that for the iilliterate and undergraduate men (*p* = 0.02), no significant difference was found in this regard in the participant women (p = 0.24).

Mean scores for the QoL in women who took oral drugs was significantly higher than that for Insulin users (p = 0.02), this score was also higher in the men with HbA1c < 7 in comparison to individuals with HbA1c > 7 group (*p* = 0.006).

Mean scores of QoL in men (p = 0.02) and VAS score in all subjects (*p* = 0.003) with a history of diabetes less than 10 years was significantly higher than subjects with a history of diabetes higher than 10 years (Table 2).

As illustrated in Table 3, each reported problem (percentage) by the patients was assessed in terms of theire demographic and clinical characteristics. In the mobility dimension, more problems were observed in the age group 50–60 years old (40.3%), housewives (42.1%), subjects with more than 10 years of DM history (44.1%) and those with nephropathy (46.4%) and neuropathy (62.5%). In the self-care dimension, more problems were reported by the housewives (21.4%), subjects with more than 10 years of DM history (25.2%), and in rural area residents (38.1%). In the usual activities dimension, more problems declared in the age group 50–60 years old (28.8%), subjects with more than 10 years of DM history (28.2%), those with nephropathy (23.6%), neuropathy (37.5%) and a hospitalization history (25%). In the pain/discomfort dimension, more problems were observed in women (56.5%), housewives (57.2%), and patients with a history of nephropathy (64.3%) and neuropathy (68.8%). Finally, in the anxiety/depression dimension, more problems reported by women (53.9%), housewives (52.4%), and rural residents (66.7%) in comparison to men, employed subjects and urban residents.

**Table 3:** The abundance of problems (percentage) reported by the patients in terms of theire demographic and clinical characteristics

variable		Mobility		Self Care		Usual Activities		Pain / Discomfort		Anxiety / Depression	
		% of any problem	P value	% of any problem	P value	% of any problem	P value	% of any problem	P value	% of any problem	P value
sex	male	23	0.001	9.8	0.002	14.9	0.07	31.1	< 0.001	28.7	< 0.001
	female	42.1		24.2		23.6		56.5		53.9	
Age group	≤50y	16.1	0.005	12.5	0.31	3.6	0.001	32.1	0.10	37.5	0.53
	51-60y	40.3		21.8		28.8		47.9		43.7	
	>60y	36.8		17.6		19.2		48		46.4	
Job	Employed	18.2	0.002	6.5	0.007	11.8	0.10	26	< 0.001	24.7	< 0.001
	housewife	42.1		21.4		22.1		57.2		52.4	
	others	35.9		24.4		24.4		41		46.2	
Education	Illiterate	43.1	0.29	20.7	0.17	20.7	0.54	53.4	0.29	48.3	0.42
	undergraduate	32		14.4		17.8		44.4		45.1	
	postgraduate	32.6		23.6		23.6		40.4		38.2	
Residency	Urban	33.3	0.18	16.8	0.01	18.7	0.03	44.1	0.24	41.9	0.02
	rural	47.6		38.1		38.1		57.1		66.7	
treatment	Oral drug	31.3	0.06	14.5	0.005	17.6	0.05	42.7	0.13	43.2	0.69
	insulin	43.1		29.2		28.2		52.8		45.8	
HbA1C	< 7	24.6	0.08	10.5	0.09	16.1	0.40	42.1	0.62	43.9	0.97
	≥7	36.6		20.2		21		45.7		43.6	
duration	<10y	28.6	0.006	14.3	0.01	15.3	0.008	43.9	0.62	41.3	0.27
	≥10y	44.1		25.2		28.2		46.8		47.7	
Nephropathy	yes	46.4	0.03	25	0.15	23.6	0.46	64.3	0.001	53.6	0.09
	no	31.6		16.8		19.3		40.6		41.4	
Retinopathy	yes	41	0.13	20.5	0.55	24.1	0.33	50.6	0.22	43.4	0.95
	no	31.8		17.5		18.5		42.9		43.8	
Neuropathy	yes	62.5	< 0.001	25	0.30	37.5	0.009	68.8	0.004	53.1	0.25
	no	31		17.5		18		42.2		42.5	
IHD	yes	40	0.41	22.9	0.48	22.9	0.71	47.1	0.61	45.7	0.63
	no	32.8		17		19.3		44.5		43.2	
Diabetes related hospitalization	yes	42.1	0.008	20.7	0.31	25	0.04	50	0.10	48.6	0.10
	no	27.5		16.3		15.7		40.6		39.4	

### Regression analysis

In logistic regression model, after dichotomizing the dependent variable in each domain, the relevant variables (as mentioned in method section, statistical analysis) entered into regression model. To follow the results easier, only those variables which exerted a significant relationship with any domain from EQ-5D-5 L were reported in Table 4. Indeed sex (OR = 2.8, CI:1.6–5), duration of diabetes (OR = 1.7, CI:1–2.9) and neuropathy (OR = 2.4, CI:1.5–5) in mobility dimension; sex (OR = 3.9, CI:1.7–8.8), job (OR = 2.7, CI:1.4–5) and residence place (OR = 4.2, CI:1.5–11.6) in the self-care dimension; duration of diabetes (OR = 2, CI:1.1–3.7) and residency (OR = 2.8, CI:1.1–7.6) in the usual activities; sex (OR = 2.9, CI:1.7–4.9), job (OR = 2.6, CI:1.3–4.9) and nephropathy (OR = 2.6, CI:1.3–4.9) in the pain/discomfort dimension; and sex (OR = 3.1, CI:1.8–5.2), job (OR = 1.7, CI:1.2–2.5) and place of residence (OR = 2.8,CI:1–7.5) in the anxiety/depression dimension showed a significant relationship with the QoL.

**Table 4:** Independent association of relevant variables with QoL dimensions in diabetic patients

	characteristic	B	SE	P value	Odds ratio	CI 95(Exp B)
Mobility	sex	1.04	0.28	< 0.001	2.84	1.61–5.01
	duration	0.54	0.27	0.04	1.72	1.00–2.94
	Neuropathy	0.88	0.42	0.03	2.43	1.06–5.55
Self Care	sex	1.36	0.41	0.001	3.93	1.75–8.82
	job	1.00	0.31	0.001	2.73	1.47–5.07
	Residency	1.43	0.51	0.006	4.20	1.52–11.60
Usual Activities	duration	0.71	0.31	0.02	2.04	1.11–3.77
	Residency	1.05	0.49	0.03	2.87	1.08–7.60
Pain / Discomfort	sex	1.08	0.26	< 0.001	2.96	1.77–4.95
	Nephropathy	0.95	0.33	0.004	2.60	1.35–4.99
Anxiety / Depression	sex	1.14	0.26	< 0.001	3.13	1.87–5.25
	job	0.56	0.18	0.002	1.76	1.22–2.53
	Residency	1.03	0.50	0.04	2.80	1.03–7.57

## Discussion

In the present study which aimed to assess the QoL in type 2 diabetic patients using the EQ-5D-5 L questionnaire, the mean score for QoL and VAS scale were 0.89 ± 0.13 and 65.22 ± 9.32, respectively. In Javanbakht study, the mean score of QoL was 0.7 (in the interval of 0.69–0.71) and VAS score was 56.8 (in the interval of 56.15–57.5) [20]. Similar studies using EQ-5D in Japan, Norway, and Korea reported a QoL score of 0.84, 0.85 and 0.91, respectively [13, 21, 22]. Considering the fact that EQ-5Dvalue sets for each country could be different, QoL is affected by various socio-economic factors and indicators such as age, DM history and complications. This notion should be considered and assessing the results should be interpreted cautiously when comparing the QoL scores. In this context, one of the challenging issues in the developing countries like Iran is that many patients usually are not aware from their illness until the onset of the complications [20, 23].

Our finding showed that most patients did not suffer from any problem or reported mild problems in some dimensions. It was also evident that moderate and severe issues were more common in the dimensions like anxiety/depression, pain/discomfort, and mobility. In this area, numerous studies reported that pain and depression as the major complaints by the patients [20, 22, 24]. In a study by Solli in 2010, pain and depression were considered as the major complaints for the diabetic patients [22]. Javanbakht et al., in 2012 also reported that challenges for DM patients were mostly common in the pain and depression dimensions [20]. Pain and mobility were the most predominant complaints of diabetic patients reported by Sakamaki et al., [21]. In parallel with different studies conducted in this field, our study also confirmed that the majority of patients were complaining from moderate to severe problems in depression, pain and mobility dimensions.

In the present study, mean scores for QoL and VAS scale were significantly higher in men, urban residents and employed patients. This could be due to the higher level of activities and the opportunity for having a better socio-economic status for the populations living in the urban areas, working people and men when compared with the unemployed patients, rural residents and women, especially in the developing countries such as Iran. In addition, since women in comparison to men showed a higher tendency for expressing health-related problems, it seems that they have a lower QoL score, which is similar and consistent with the findings of previous studies [13, 21, 25, 26].

After entering and analyzing the variables related to the regression model, it is evident that the gender variable showed a significant relationship with all dimensions of QoL, with the exception of the usual activities, so that women in the mentioned dimensions had lower QoL than men. Also, the highest correlation was found among place of residence and sex with self-care dimension. In Javanbakht study [20], individuals living in bigger cities had lower QoL than those in small cities in the self-care dimension.

Our finding also suggested that the mean score for QoL in the older age groups was lower from younger groups. Indeed, most of the complaints and problems were reported by patients who belonged to people older than 50 years. Moreover their complaints were about usual activities and mobility which were consistent with other reported studies [13, 20, 25]. Conversely, in studies such as O’Reilly et al., [27] the QoL scores increased with age, which could be due to different economic and social conditions in different societies.

Our study showed that patients with higher level of education possess a better QoL score. It should be noted that no significant difference was found in the QoL score for men with different levels of education; however, it was significantly higher in the illiterate and undergraduate women when compared to the postgraduate women. VAS score was significantly associated with higher education level for men, this information was also in line with other studies which shown positive effects on improving the QoL for DM patients. It could be due to better understanding of the disease and the proper and timely pursuit for better disease control and treatment [22, 25]. In illiterate women due to lack of enough knowledge on the disease and its health consequences, it exerted a lower impact on their QoL.

In terms of the treatment type and the mean scores for the QoL and the VAS scale, our finding suggest that patients treated with insulin had significantly lower mean scores for QoL compared with individuals who received oral treatment. It was even more evident in the self-care dimension which insulin user patients reported more problems in comparison to the oral drug users. In this regard and considering the fact that insulin is used as the last resort when the oral therapy is ineffective in patients with type 2 diabetes, longer periods of diabetes are expected in insulin-dependent patients resulting in a direct negative impact on the patients’ QoL [4, 13]. This result is consistent with Redekop et al., study suggesting that insulin-dependent diabetes patients had lower QoL in Germany [28]. Conversely, in studies such as Bradley et.al [29], none of the treatments showed significant associations with EQ-VAS health status.

The results of our study showed that patients with a history of hospitalization had significantly lower QoL and VAS scores. They also reported more problems in terms of mobility and their usual activities. Due to weakness of the immune system, diabetic patients are more vulnerable to various types of infections, and on the other hand, the chance of acute and chronic complications is high due to the illness’ nature and the lack of proper control for DM [30]. The history of hospitalization may be indicative of inappropriate control for the disease and its complications which could justify a lower quality of life in this group of patients. Numerous studies in this regard suggested that diabetic patients with a hospitalization history have been associated with lower QoL [21, 22].

In the present study, a history of longer than 10 years of DM and the presence of chronic complications including neuropathy and nephropathy were significantly associated with a decreased level of QoL and VAS scale. More significant problems were observed in patients with nephropathy in terms of mobility and pain, and individuals with neuropathy in terms of mobility, pain and routine activities. Similar studies have shown the lower QoL scores in patients with history of hospitalization, history of over 10 years with DM and the presence of chronic complications [20, 22, 25, 27, 31], which was in line with our findings.

Although in our study, patients with HbA1c level below 7 had a higher score of the QoL than those with the level greater than 7, it was only statistically significant in men. Considering the direct correlation among DM complications and the proper control for blood glucose level [30] and the fact that HbA1c level is indicative of DM status in the last 3 months, patients with lower HbA1c level are expected to have a better QoL with lower complications.

In this study, we have encountered several limitations as follow:

We have selected the participants from one diabetic clinic which were not included all diabetic patients in the city, therefore the results cannot be representative for all the diabetic patients in the city, which is one of the limitations of the study.

We collected the patients’ information from their profile and medical records which were previously gathered and recorded. These data include the diabetes treatment, laboratory values, presence of complications, type of complication (nephropathy, retinopathy and neuropathy), hospitalization history due to diabetes and history of IHD.

Although diabetes complications were related to individuals’ HRQoL, we did not assess all diabetic complications which influence the HRQoL. Furthermore, since this is a cross-sectional study, the observed associations are not necessarily causal. The absence of a locally appropriate set of values in our country was other limitation.

We did not also follow the PROMs guidelines for translation; this could be the other limitation for the present study.

## Conclusion

The quality of life for the patients with type-2 diabetes is affected by numerous factors such as sex, occupation, DM history and the presence of complications including neuropathy and nephropathy. Therefore, much more attention should be paid toward the key determinants of HRQoL to identify and implement the appropriate policies for achieving better management for DM and ultimately improving the QoL for diabetic patients in this region.

## Data Availability

Please contact the corresponding author for data requests.

## Abbreviations

A/D: Anxiety/depression
DM: Diabetes mellitus
EQ-5D-5 L: EuroQol five dimensions scale 5 levels
IHD: Ischemic heart disease
MO: Mobility
P/D: Pain/discomfort
QoL: Quality of life
SC: Self-care
UA: Usual activities
VAS: Visual analogue scale
